# A national survey on assessment of knowledge, perceptions, practice, and barriers among hospital pharmacists towards medication reconciliation in United Arab Emirates

**DOI:** 10.1038/s41598-024-64605-4

**Published:** 2024-07-04

**Authors:** Alaa Farajallah, Hadzliana Zainal, Subish Palaian, Muaed Alomar

**Affiliations:** 1 Department of Clinical Sciences, College of Pharmacy and Health Sciences, Ajman University, Ajman, UAE; 2 Department of Clinical Pharmacy, School of Pharmaceutical Sciences, Universiti Sains Malaysia, Penang, Malaysia

**Keywords:** Barriers, Hospital pharmacists, Knowledge, Medication reconciliation, United Arab Emirates, Health care, Health occupations, Medical research

## Abstract

Medication reconciliation (MedRec) helps prevent medication errors. This cross-sectional, nationwide study assessed the knowledge, perceptions, practice, and barriers toward MedRec amongst hospital pharmacy practitioners in the United Arab Emirates. A total of 342 conveniently chosen stratified hospital pharmacists responded to the online survey (88.6% response rate). Mann–Whitney U test and Kruskal–Wallis test were applied at alpha = 0.05 and post hoc analysis was performed using Bonferroni test. The overall median knowledge score was 9/12 with IQR (9–11) with higher levels among clinical pharmacists (p < 0.001) and previously trained pharmacists (p < 0.001). Of the respondents, 35.09% (n = 120) practiced MedRec for fewer than five patients per week despite having a strong perception of their role in this process. The overall median perception score was 32.5/35 IQR (28–35) with higher scores among clinical pharmacists (p < 0.001) and those who attended previous training or workshops (p < 0.001). The median barrier score was 24/30 with an IQR (21–25), where lack of training and knowledge were the most common barriers. Results showed that pharmacists who did not attend previous training or workshops on MedRec had higher barrier levels than those who attended (p = 0.012). This study emphasizes the significance of tackling knowledge gaps, aligning perceptions with practice, and suggesting educational interventions.

## Introduction

The negative consequences of medication errors are acknowledged as one of the biggest threats to the global healthcare system^1,2^. Statistics from the United States (US) showed that more than 250,000 patients die every year due to medication errors^3^. In addition, medication errors lead to significant human suffering and economic losses. Multiple strategies have been implemented with varying success and undoubtedly it is possible to reduce or even prevent medication errors by developing more effective healthcare systems and implementing patient safety initiatives^4^. One such initiative to minimize medication errors is medication reconciliation (MedRec), which is acknowledged as preventing possible medication errors and enhancing the accuracy of medication lists^5^. In definition, MedRec is “the process of creating the most accurate list possible of all medications a patient is taking—including drug name, dosage, frequency, and route—and comparing that list against the physician’s admission, transfer, and/or discharge orders, to provide correct medications to the patient at all transition points within the hospital”^6^.

Medication reconciliation is performed by physicians, nurses, and pharmacists. It is well reported that pharmacists can have a significant impact on enhancing the safety of medication by contributing to the MedRec process and correcting any medication discrepancies^7^. Many published data show that pharmacists are specially prepared to lead interdisciplinary efforts and build an efficient MedRec process in hospitals and across health systems because of their specific knowledge, skills, and talents^7–10^. The American Society of Health-System Pharmacists (ASHP) established a guidance document for pharmacist’s involvement in the MedRec process^11^. Globally, MedRec is practiced with varying degrees in which Western countries have better MedRec practices. Reviewing the literature indicated a significant divergence in the current practice of MedRec within Gulf Cooperation Council (GCC) countries which can be due to variable policies set by both the health ministries and individual hospitals within the countries^12^.

In the United Arab Emirates (UAE), the practice of MedRec is less common, and in the past, the Department of Health Services in the UAE has adopted several plans to enhance medication therapy management Services^13^. In 2022, the Department of Health (DOH) in Abu Dhabi issued a Standard for Primary Healthcare Services in the Emirate of Abu Dhabi, which emphasized the necessity for healthcare facilities to implement suitable policies and procedures for MedRec^14^. In February 2024, the same department released an article addressing the scope of practice for pharmacists in hospital settings^15^. It emphasized that MedRec is one of the responsibilities of pharmacists both in outpatient and inpatient care. Furthermore, it stressed the importance of clinical pharmacists collaborating with physicians during admission, transitions in care, and discharge for effective MedRec. However, there is a dearth of publications addressing the specific role of hospital pharmacists in this process. For instance, two publications from Abu Dhabi Emirate, the capital of the UAE, focused on enhancing MedRec practices in their facilities but did not determine the role of pharmacists. Instead, they mentioned MedRec is an interdisciplinary task involving physicians, nurses, and pharmacists^16,17^.

Involvement of community pharmacists in this process in UAE is very limited due to many barriers including lack of time and staff; the complex nature of the process; and barriers related to the patients^18^. An interventional study conducted across 25 community pharmacies in the UAE underscored the urgent necessity for interventions like pharmacist-patient-centered medication reconciliation to overcome these obstacles^19^. Added to this, there has been no research conducted in UAE to explore the knowledge, perception, practice, and barriers related to MedRec process in UAE.

Understanding the knowledge of pharmacists can provide valuable information on specific knowledge gaps within the UAE pharmacists and thus give opportunity for future researchers to develop education modules to minimize the knowledge gap. The existing literature highlighted differences in pharmacists’ perspectives concerning MedRec and whether they are capable of intervening in this process^20,21^. Identifying the current perceptions of hospital pharmacists regarding MedRec will aid in devising strategies to enhance their perspectives and engagement in this critical process. Moreover, to make the pharmacist-led medication reconciliation procedure more practicable, it is necessary to identify the physical obstacles that prevent its implementation in the UAE. As mentioned earlier, currently there are no studies conducted in the UAE to address these aspects, therefore this study was proposed with the objectives of assessing the knowledge and perceptions of hospital pharmacists regarding MedRec; evaluating the existing practices of MedRec carried out by hospital pharmacists, and identifying and addressing the obstacles that hinder the effective implementation of MedRec.

## Methodology

### Study design

This study is a cross-sectional survey performed to evaluate knowledge, practice, perceptions and barriers of MedRec among pharmacists working in the UAE hospitals. This manuscript adheres to and conforms with “The Strengthening the Reporting of Observational Studies in Epidemiology” (STROBE) statement^22^.

### Study timeline

The study was conducted from 20 March to 20 June 2023.

### Ethical approval

Ethical approval of this research has been obtained from the following research committees: 1. Research Ethics Committee of Ajman University (Reference Number: Reference number: P-F-H-11-Mar), 2. Ministry of Health and Prevention Research Ethics Committee (Approval Reference No: MOHAP/DXB-REC/ JJA/No. 60/ 2022), 3. Dubai Scientific Research Ethics Committee (Approval reference NO: DSREC-SR-10/2022_01), 4. Abu Dhabi Health Research and Technology Ethics Committee (Approval reference NO: DOH/CVDC/2022/1637), and 5. Human Research Ethics Committee Universiti Sains Malaysia (JEPeM Code: USM/JEPeM/22090577).

Informed consent was obtained from all participants prior to enrolling in the research. The participation was purely voluntary. The researchers maintained anonymity and confidentiality of participants’ responses. All the requirements specified by the ethical approving bodies were strictly followed and all experiments were performed in accordance with relevant guidelines and regulations.

### Study settings

The study was conducted among pharmacists working in UAE hospitals from all seven Emirates of UAE.

### Target population

Hospital pharmacists working in UAE hospitals and aged between 22 and 60 years old were included. The pharmacists working in community pharmacies, pharmacists working in places other than hospitals such as: drug stores, pharmaceutical companies, universities and pharmacists not willing to sign the consent form were excluded from the research.

### Sample size calculation

A sample size of 385 was determined using Cochran’s formula^23^. In this study, the population size consisted of the total number of pharmacists working in UAE hospitals. There are no published data on the total number of pharmacists working in UAE hospitals specifically. However, based on the statistics in 2020, there are 11,153 total pharmacists working in both community and hospital pharmacies in the UAE (5518 males and 5635 females)^24^ and some publications mention about 1300 pharmacists working in community pharmacies^25^. By estimating the total number of hospital pharmacists in UAE, it was calculated as 9853 pharmacists. The sample size is determined using the Cochran formula as outlined below:

$${n}_{0=\frac{{Z}^{2}pq}{{e}^{2}}.}$$

For the calculation of the required sample size (n_0_), the following parameters are used: Z, which is set at 1.96 to achieve a 95% confidence interval (CI), and a marginal error of 0.05. The expected proportion (p) is assumed to be 50%, and precision (e) is set at 5%. Consequently, we obtained ((1.96)^2^ (0.5) (0.5))/(0.05)^2^ = 385.

### Sampling method

In this research, stratified convenient sampling technique was used in collecting data. The population is hospital pharmacists in UAE (N = 9853). The ‘working Emirate’ was selected as stratification in this study, so there were seven strata. The next step required listing the population which means having an access to all hospital pharmacists’ records working in UAE. In fact, the researchers did not have access to these records, instead, they listed the details of all hospitals in UAE and this was taken from the yellow pages. The hospital details were added to an excel sheet, then the population was listed according to the chosen stratification. For this step, a consecutive number was given to each hospital in each stratum. As a result, authors arrived at seven lists as the following: Abu-Dhabi hospitals, Dubai hospitals, Sharjah hospitals, Ajman hospitals, Ras Al-Khaima hospitals, Fujairah hospitals, and Umm Al-Qawain hospitals.

The following step was to choose the sample size (n) which was calculated using Cochran formula (n = 385). Then, the proportionate stratification was calculated by multiplying the intended sample size (n) by the proportion of units within each stratum, therefore, the number of hospital pharmacists in each Emirate has to be known. Since this number is unknown, the researchers tried to figure it out by doing the following: first, the published data about the percentage of the UAE population in each Emirate was searched^26^. Then, the total number of hospital pharmacists in UAE (9853) was multiplied by the proportion of the population in each Emirate. The resulted number for each Emirate yielded a summation of 7853. Calculation was made to reach this summation to 9853 and the number of hospital pharmacists in each Emirate was defined as shown in Table 1.

**Table 1:** Distribution of hospital pharmacists among the seven Emirates in UAE.

Name of Emirate	P	N × P	N × P*	p̂	n x p̂
Abu-Dhabi	0.1540	1518	1904	0.19	74
Dubai	0.3488	3437	4312	0.437	167
Sharjah	0.1786	1760	2207	0.224	87
Ajman	0.0504	497	623	0.0633	25
Ras Al-Khaima	0.0345	340	427	0.0433	17
Fujairah	0.0256	253	318	0.0322	13
Um Al-Qawain	0.0049	49	2	0.0061	2
Total	–	7854	9853	–	385

*n* sample size, *N* population size, *P* population in each Emirate, $\hat {p}$ Proportion of hospital pharmacists in each Emirate, *n* ×  $\hat {p}$ estimated number of hospital pharmacists in each Emirate.

*The calculation was done to adjust the summation of hospital pharmacists to 9853.

The last step was applying a simple random sampling technique for the selection of the listed hospitals. Once the hospitals had been selected, their affiliated pharmacists were approached via the LinkedIn platform.

### Data collection instrument

The study questionnaire had five parts; 1. Demography, 2. knowledge (self- developed), 3. perception, 4. practice and 5. barriers (all later three adapted from a Jordanian study)^27^ after obtaining permission. A thorough literature review was done to develop knowledge questions with extensive discussions between the co-authors^28–31^.

#### Knowledge part of the questionnaire

The knowledge questions are built based on four main domains (What, Who, Why and How) as the following: What is MedRec, 3 questions; objectives of MedRec, 2 questions; Who can do MedRec, 2 questions and How to do MedRec, 5 questions. The knowledge about MedRec was assessed by asking the participants 12 multiple-choice questions. The researchers preferred to ask multiple choice questions rather than “Yes”, “No” questions to assess the real knowledge of participants about this comprehensive process.

The content validation was done by three experts. The panel of experts comprised two clinical pharmacists holding Board Certification as Pharmacotherapy Specialists in the UAE and an academician in the University of Central Lancashire in UK. Their suggestions and comments were considered in updating the knowledge questions as the following: adding “I do not know” option to all knowledge questions and changing the word “correct” to “appropriate” in one of the answer responses of question 9.

After that, face validity was done by distributing the questionnaire to five hospital pharmacists working in different Emirates. They were requested to fill out the questionnaire and give their feedback on its clarity, relevance, appropriateness, and adequacy. Their comments were positive towards the questions and the questionnaire was finalized with 12 items.

#### Assembly of the final tool

The final study tool had five components. In *Part I*, there were nine questions related to participants’ sociodemographic information, followed by five general questions. *Part II* involved a knowledge assessment that encompassed 12 items and was evaluated through multiple-choice questions*.* For *Part III*, the current practice of hospital pharmacists on MedRec was evaluated by asking four questions. In *Part IV*, the focus was on pharmacists’ perceptions of MedRec, which included seven items. Pharmacists were requested to indicate their level of agreement using a 5-point Likert scale, ranging from 5 for “strongly agree” to 1 for “strongly disagree”. Part *V* consisted of six questions related to Pharmacists’ perceived barriers towards MedRec where a 5-point Likert scale was used.

### Questionnaire scoring

#### Knowledge scoring

The 12 knowledge questions about MedRec were determined by asking multiple-choice questions with only one correct answer. The phrase “I do not know” was added as an option to select for all knowledge questions. The correct answer scored (1) while the incorrect answers including “I do not know” answer scored (0.0).

#### Practice scoring

Pharmacists’ practice was assessed by asking three questions in a form of “Yes”, and “No” questions while the fourth question was a multiple-choice question with four available options.

#### Perceptions and perceived barriers scoring

For the perceptions and perceived barriers parts, 7 and 6 questions were asked respectively. These items were rated on a 5-point Likert scale (1 = “strongly disagree,” 2 = “disagree,” 3 = “neutral,” 4 = “agree,” 5 = “strongly agree”). Analysis involved counting the total responses for each level of sentiment (Strongly Disagree, Disagree, Neutral, Agree, Strongly Agree) for each question.

### Data collection

The pharmacists were accessed via the LinkedIn platform where screening of their affiliation and designation was done by the researcher and those who are eligible for the study received the participants’ information sheet and the consent form. Those willing to participate and signed the consent form received a google link to fill in by answering the survey questions.

### Pilot testing

A pilot study was conducted during 1st March till 15th March 2023 by distributing the questionnaire to 17 hospital pharmacists whose data were subsequently omitted from the final analysis of the study. The purpose of the pilot study was to check the feasibility of the study and assess the reliability of the study tool. Using SPSS software version 28.0, Cronbach’s alpha (α) was computed to evaluate internal consistency and scale reliability. The calculated Cronbach’s alpha (α) value was 0.781, which means that the items have relatively high internal consistency^32^.

### Data analysis

The data collected through the Google survey was imported into IBM Statistical Package for the Social Sciences (SPSS) Version: 28.0.0.0 (190). Data were analyzed quantitatively using descriptive statistics (mean, standard deviation (SD), median, range, frequency and percentage) in addition to inferential statistics. Results of sociodemographic data in addition to training and practice questions were reported as frequencies and percentages. Results of perceptions and perceived barriers were described as the percentage of pharmacists who expressed varying degrees of agreement with each statement, ranging from strongly agree to strongly disagree.

All quantitative scales for knowledge, perception, and barriers are normalized on a 100-point scale from 0 to 100. The test of normality for knowledge, perception, and barriers scales was done using the Shapiro–Wilk normality test. Since all scales are not normally distributed (p value = 0.000), non-parametric tests are considered for relations. Mann and Whitney U test for comparing two independent groups (gender, country of graduation, clinical pharmacist and previous training on MedRec) whereas the Kruskal Wallis test was used when dealing with multiple independent groups, exceeding two (age, years of experience, education level, and working Emirate). For groups with significant relations, post hoc analysis was done using pairwise comparison test and Bonferroni was applied. The correlation of the main scales (total knowledge scale and total perception scale; total knowledge scale and total perception scale; total perception scale and total barriers scale) was done using Spearman’s rho test. A *moderate* correlation was considered if the correlation coefficient is between ± 0.4 to ± 0.6 while it was considered *weak* if the correlation coefficient is between ± 0.1 and ± 0.3^33^. Throughout the analysis, a p-value of < 0.05 was considered statistically significant.

## Results

Hospital pharmacists included in the study are working in the seven Emirates: Abu Dhabi, Dubai, Sharjah, Ajman, Ras AlKhaima, Um Al-Qawain and Fujairah. The survey was distributed to 386 hospital pharmacists working in the UAE and of those, 342 filled out the questionnaire giving an overall response rate of 88.6%. The total number of participants was 88.8% of the calculated sample size.

### Sociodemographic profile of participants

The socio-demographic characteristics of the participants are presented in Table 2. Participants were mostly females (54.7%) whereas the percentage of males was 45.3%. The mean age for the sample group is 31.38 with 95% CI between (30.75–31.94) while the mean years of experience is 5.47 with 95% CI between (5–6). Most of the participants are Bachelor’s degree holders (54.7%) whereas (29.24%) are clinical pharmacists. Among the participants, 141 (41.20%) are working in outpatient pharmacies.

**Table 2:** Demographic characteristics of participants (n = 342).

Study variables	Frequency (%)
Gender*	
Male	155 (45.32)
Female	187 (54.68)
Age (years)* [31.38 ± 5.53]	
≥ 20–< 30	155 (45.32)
≥ 30–< 40	143 (41.81)
≥ 40–< 50	34 (9.94)
Country of graduation*	
Arab states	226 (69.1)
Asia and Pacific	98 (29.9)
The Americas	2 (0.6)
Europe	1 (0.3)
Years of experience* [5.47 ± 4.55]	
≥ 0–< 5	188 (54.97)
≥ 5–< 10	82 (23.98)
≥ 10–< 15	64 (18.71)
Qualification	
Bachelor	187 (54.70)
Master and Pharm D	150 (43.90)
PhD	5 (1.50)
Hospital type	
Semi-government hospital	65 (19.01)
Private non-teaching hospital	121 (35.38)
Private teaching hospital	58 (16.96)
Government hospital	98 (28.65)
Site of work	
Outpatient pharmacy	141 (41.20)
Inpatient pharmacy	74 (21.60)
Both inpatient and outpatient pharmacy	1 (0.30)
Pharmacy on duty	22 (6.40)
Emergency pharmacy	8 (2.30)
Working in all the above-mentioned sites	85 (24.90)
Working in other sites not mentioned above	11 (3.20)
Clinical pharmacist	
Yes	100 (29.24)
No	242 (70.76)
In which Emirate are you working	
Abu-Dhabi	127 (37.13)
Dubai	113 (33.04)
Northern Emirates** (Sharjah, Ajman, Um-AlQawain, Ras Al-Khaima, Al-Fujairah)	102 (29.82)

*Pharm D* Doctor of Pharmacy, *PhD* Doctor of Philosophy.

*Indicates missing values.

**Pharmacists participated from each Northern Emirate as the following: Sharjah n = 42; Ajman n = 33; Um-AlQawain n = 5; Ras Al-Khaima n = 14; Al-Fujairah n = 8.

### Pharmacists’ skills and training about medication reconciliation

More than 90% of the respondents stated they have enough skills to take the best possible medication history from patients. Similarly, the majority reported their confidence in having enough skills to apply MedRec during patients’ transition of care (80.7%). When asked about their previous training or study on MedRec, more than half of the participants denied studying a related course in their university (52.92%) and four-fifths of participants agreed on the need for more training programs about MedRec (Table 3).

**Table 3:** Pharmacists’ skills and training about medication reconciliation.

Question	Response	n = 342 (%)
Do you have enough confidence skills on “taking the best possible medication history”?	Yes	317 (92.69)
	No	25 (7.31)
Do you have enough confidence skills in applying medication reconciliation during patients’ admission, transfer, and discharge?	Yes	276 (80.70)
	No	66 (19.30)
Did you study a course or did training about medication reconciliation during your university study?	Yes	161 (47.08)
	No	181 (52.92)
Did you receive training or attended a workshop on medication reconciliation during my working experience?	Yes	193 (56.43)
	No	149 (43.57)
I feel I need more training programs about medication reconciliation	Yes	276 (80.70)
	No	66 (19.30)

### Pharmacists’ knowledge about medication reconciliation

The overall median knowledge score was 9 with IQR (9–11) and the maximum possible knowledge score was 12. The majority of participants could define MedRec correctly (81.9%); identify its goal (91.2%) and know which medications to ask during the process (86%). When focusing on the technique of conducting the process, the percentages of correct answers fluctuated as only 54.7% know that medication discrepancies can be intentional and unintentional and 58.5% understand how to achieve the “best possible medication history” while 66.4% are aware of which stage MedRec can be applied. Participants’ correct, incorrect and “I do not know” responses to each knowledge question are shown in Table 4.

**Table 4:** Knowledge about medication reconciliation among hospital pharmacists in UAE.

Knowledge questions	Matching knowledge questions with domains	Correct (%)	Incorrect (%)	“I do not know” (%)
The term “Medication Reconciliation” means	What	280 (81.9)	38 (11.1)	24 (7%)
Which of the following medications are important to ask when conducting medication reconciliation?	How	294 (86)	20 (5.8)	28 (8.2)
Collecting the best possible medication history should include	How	299 (87.4)	22 (6.5)	21 (6.1)
The “Best Possible Medication History” can be achieved by	How	200 (58.5)	121(35.4)	21 (6.1)
While taking the “best possible medication history” from patients, the most suitable healthcare provider is	Who	121 (35.4)	211 (61.7)	10 (2.9)
Medication discrepancies can be divided into intentional and unintentional discrepancies	What	187 (54.7)	24 (7)	131 (38.3)
Which of the following issues is not categorized as medication error?	What	269 (78.7)	30 (8.7)	43 (12.6)
The goal of medication reconciliation is to:	Why	312 (91.2)	10 (3)	20 (5.8)
Medication reconciliation is important for patient safety because?	Why	297 (86.8)	25 (7.4)	20 (5.8)
Who is the responsible healthcare professional to report a medication error to the concerned authority?	Who	274 (80.1)	53 (15.5)	15 (4.4)
Medication reconciliation can be implemented in which of the following stage(s)?	How	227 (66.4)	90 (26.3)	25 (7.3)
If a patient is not responding well to your queries while you are obtaining his/her medication history, what can you do?	How	257 (75.1)	63 (18.5)	22 (6.4)

### Pharmacists’ current practice of MedRec

The current practice of MedRec was assessed by asking 4 questions. Most of the participants (83.33%) are aware if there is a MedRec policy in their hospital. The majority (72.51%) answered implementing MedRec is a part of their job description and they have to apply it to all admitted patients (74.56%). When asked “on average, for how many patients do you implement medication reconciliation in your pharmacy per week?”, 35.09% selected less than 5 patients; 23.68% answered between 5 and 9 patients; 11.7% stated from 10 to 14 patients and 29.53% reported more than 15 patients (Table 5).

**Table 5:** Current practice of medication reconciliation in hospitals.

Question	Response	n = 342 (%)
Are you aware if there is a medication reconciliation policy in your hospital?	Yes	285 (83.33)
	No	57 (16.67)
Do you have to implement medication reconciliation to admitted patient in your job description?	Yes	248 (72.51)
	No	94 (27.49)
Do you have to implement medication reconciliation to all admitted patients?	Yes	255 (74.56)
	No	87 (25.44)
On average, for how many admitted patients do you implement medication reconciliation in your pharmacy per week?	More than 15	101 (29.53)
	Between 10 and 14	40 (11.70)
	Between 5 and 9	81 (23.68)
	Less than 5	120 (35.09)

### Pharmacists’ perception of medication reconciliation

The test of normality the for perception scale was not normally distributed with an overall median perception score was 32.5 IQR (28–35) and the maximum possible perception score is 35. The Perception of MedRec among hospital pharmacists was assessed by asking 7 questions and the results of each question are shown in Table 6. The maximum number of participants (97.96%) perceived that pharmacists can help in assessing the appropriateness of medications for patients.

**Table 6:** Pharmacists’ perceptions towards implementing MedRec.

Perception questions	Strongly disagree (1)		Disagree (2)		Neutral (3)		Agree (4)		Strongly agree (5)		Median (IQR)
	Frequency	(%)	Frequency	(%)	Frequency	(%)	Frequency	(%)	Frequency	(%)	
Implementing medication reconciliation should be provided to all patients	1	0.29	16	4.68	27	7.89	123	35.96	175	51.17	5 (4–5)
Pharmacists can help in assessing the appropriateness of medications or patients	1	0.29	0	0.00	6	1.75	136	39.77	199	58.19	5 (4–5)
I am confident that I can provide support and advice for health care providers about the medications prescribed for patients	2	0.58	3	0.88	18	5.26	147	42.98	172	50.29	5 (4–5)
Pharmacists should be consulted for advice when medical staff are considering discontinuing or prescribing medications	1	0.29	1	0.29	21	6.14	151	44.15	168	49.12	4 (4–5)
Pharmacists can counsel and educate patients, their families and the nursing staff about medications	1	0.29	0	0.00	11	3.22	112	32.75	218	63.74	5 (4–5)
Pharmacists must be integral members of the healthcare team providing services for patients	1	0.29	0	0.00	8	2.34	114	33.33	219	64.04	5 (4–5)
Pharmacists can help the doctor in identifying suitable formulations of medications	1	0.29	1	0.29	7	2.05	105	30.70	228	66.67	5 (4–5)

*IQR* interquartile range.

### Pharmacists’ perceived barriers on implementing medication reconciliation

Barrier scale was not normally distributed with an overall median barrier scale for all participants is 24 with IQR (21–25) and the maximum possible barrier score is 30. Pharmacists were asked 6 questions to identify the barriers that may render them from practicing MedRec. Table 7 shows participants’ responses to each question. A high agreement level among the majority of participants that the 6 listed barriers are obstacles that render them from implementing MedRec.

**Table 7:** Pharmacists’ perceived barriers towards medication reconciliation.

Perceived barrier questions	Strongly disagree		Disagree		Neutral		Agree		Strongly agree		Median (IQR)
	Frequency	(%)	Frequency	(%)	Frequency	(%)	Frequency	(%)	Frequency	(%)	
Lack of knowledge in medication reconciliation among health care providers	4	1.17	10	2.92	52	15.20	208	60.82	68	19.88	4 (4–4)
Lack of training in reconciliation among pharmacists	3	0.88	9	2.63	38	11.11	207	60.53	85	24.85	4 (4–4.25)
Lack of cooperation with health care providers regarding medication reconciliation	5	1.46	30	8.77	64	18.71	162	47.37	81	23.68	4 (4–4)
Lack of access to patients’ medical notes/shared information	10	2.92	40	11.70	59	17.25	175	51.17	58	16.96	4 (4–4)
Time barrier (pharmacists do not have time)	16	4.68	39	11.40	49	14.33	154	45.03	84	24.56	4 (4–4)
Lack of communication between pharmacists and health care providers	9	2.63	31	9.06	50	14.62	182	53.22	70	20.47	4 (4–4)

*IQR* interquartile range.

### Correlations between MedRec knowledge, perception and barriers scales

Spearman rho rank correlation test was used to show the relationships between different scale (Table 8). There is a statistically highly significant of positive correlation with moderate strength between total knowledge scale and total perception scale (p value < 0.001). On the other hand, there is a significant negative correlation with weak strength between total knowledge scale and total barrier scale (p value = 0.036).

**Table 8:** Correlations between MedRec knowledge, perception and barriers scales.

Correlations of the 3 scales (knowledge, perception, barriers)	Spearman’s rho	p value
Total knowledge scale and total perception scale	0.319	< 0.001*
Total knowledge scale and total barriers scale	− 0.113	0.036*
Total perception scale and total barriers scale	0.065	0.233

*p value < 0.05 indicates statistically significant correlations.

### Influence of demographic factors on respondents’ knowledge, perception and barriers

Demographic Factors that affect hospital pharmacists’ knowledge of MedRec were evaluated using the Mann and Whitney U test (for 2 groups) and the Kruskal Wallis test (for > 2 groups). Post hoc analysis was done by applying Bonferroni test whenever there are significant relations between groups.

#### Knowledge

The results showed that clinical pharmacists have better knowledge scores than non-clinical pharmacists as the p-value is highly significant (p value < 0.001), also those with master or pharm D and PhD qualifications showed a highly significant difference in their knowledge than bachelor holders (p value < 0.001). Moreover, pharmacists who took training on MedRec previously or studied a course in the university have higher knowledge compared to those who did not (p value < 0.001). Years of experience is also a factor that affects the knowledge of the participants as those with more experience (≥ 5–< 10; ≥ 10–< 15) have higher knowledge than those with fewer experience years (≥ 0–< 5), where the p value < 0.001. Similarly, the results indicate that as the age of participants increases, their knowledge also increases (p value < 0.001). On the other hand, gender, and country of graduation do not influence on knowledge (Tables 9, 10).

**Table 9:** Participants’ knowledge, perception, and barriers towards MedRec vs demographic variables (two independent groups) using Mann Whitney U test.

Demographics	Groups	n	Knowledge scale			Perception scale			Barrier scale		
			Median	IQR	p value	Median	IQR	p value	Median	IQR	p value
Gender	Male	155	9	8–11	0.991	33	28–35	0.870	24	21–24	0.132
	Female	187	9	8–11		32	28–35		24	21–25	
Country of graduation	Arab States	226	9	7–11	0.862	32	28–35	0.490	24	21–25	0.888
	Asia and Pacific	98	9	8–10.25		33	28–35		24	20–25	
Clinical Pharmacist	Yes	100	10.5	9–11	< 0.001*	34	31–35	< 0.001*	24	20–25	0.345
	No	242	9	7–10		31	28–35		24	21.75–25	
Previous course/training on MedRec in University	Yes	161	10	8.5–11	< 0.001*	34	29–35	< 0.001*	24	21–26	0.340
	No	181	9	7–10		31	28–34		24	21–24	
Previous training or workshop attended during practice	Yes	193	10	8–11	< 0.001*	34	30–35	< 0.001*	24	20–25	0.012*
	No	149	8	6–10		30	28–34		24	23–25	

*IQR* interquartile range.

*p value < 0.05 was considered statistically significant.

**Table 10:** Participants’ knowledge, perception, and barriers towards MedRec vs demographic variables (more than two independent groups) using Kruskal Wallis test.

Demographics	Groups	n	Knowledge scale			Perception scale			Barrier scale		
			Median	IQR	p value	Median	IQR	p value	Median	IQR	p value
Age	≥ 20–< 30	155	9	7–10	< 0.001*	32	28–35	< 0.001*	24	21–25	0.835
	≥ 30–< 40	143	10	8–11		33	28–35		24	21–24	
	≥ 40–< 50	34	10	8.75–11		33.5	29–35		24	22–26.25	
Years of experience	≥ 0–< 5	188	9	7–10.75	< 0.001*	32	28–35	< 0.001*	24	20–25	0.100
	≥ 5–< 10	82	10	8–11		32.5	28–35		24	21–24	
	≥ 10–< 15	64	10	9–11		34	28–35		24	22–26	
Educational level	Bachelor	187	8	7–10	< 0.001*	30	28–34	< 0.001*	24	22–25	0.042*
	Master or Pharm D	150	10	9–11		34	30.75–35		23	20–25	
	PhD	5	12	9.5–12		32	29.5–33		22	17–24.5	
Working Emirates	Abu-Dhabi	127	9	8–11	0.861	31	28–35	0.004*	24	23–24	0.197
	Dubai	113	9	8–11		34	29–35		24	20–25	
	Northern Emirates	102	9	7–11		32	29–34.25		23	20–25	
Hospital type	Semi-government hospital	65	9	8–10.5	< 0.001*	29	28–34	< 0.001*	24	22–24	0.175
	Private non-teaching hospital	121	9	6–10		31	28–35		24	22–25.5	
	Private teaching hospital	58	10	8–11		34	32–35		23.5	20–26	
	Government hospital	98	10	8–11		33	29–35		24	20–25	
Working pharmacy site	Outpatient pharmacy	141	8	6–10	< 0.001*	29	28–34	< 0.001*	24	23–25	0.039*
	Inpatient pharmacy	74	10	9–11		34	31–35		23.5	19.75–25	
	Pharmacy on duty	22	10	6–11		30	28.75–32.25		22	18.75–24	
	Emergency pharmacy	8	11	10–12		30	28–34.5		24	22–24	
	Working in All mentioned sites	85	10	8–11		34	31–35		24	20–26	
	Working in other sites	11	10	7–11		33	31–35		24	19–26	

*IQR* interquartile range.

*p value < 0.05 was considered statistically significant.

Post hoc analysis using pairwise comparison showed a statistically significant difference in the total knowledge scores between the age group of [20 to below 30] and [30 to below 40] (p < 0.001), [20 to below 30] and [40 and above] (p = 0.004). There was also a statistically significant difference in the total knowledge scores between the years of experience [< 5 years] and [5–< 10 years] (p = 0.002), [< 5 years] and [≥ 10 years] (p < 0.001). A statistically significant difference was also noticed in the knowledge between the qualifications [bachelor] and [master or Pharm D] (p < 0.001), [bachelor] and [PhD] (p = 0.002). The test illustrates a statistically significant difference in the knowledge between the type of hospital [private non-teaching] and [government] (p < 0.001), [private non-teaching] and [private teaching] (p < 0.001). The working site inside the hospital is a factor that affects the knowledge as the test shows a statistically significant difference between the knowledge and site of work [outpatient pharmacy] and [inpatient pharmacy] (p < 0.001), [outpatient pharmacy] and [emergency pharmacy] (p = 0.002), [outpatient pharmacy] and [pharmacists working in all pharmacy sites in the hospital] (p = 0.001).

#### Perception

Many factors have an impact on the participants’ perception of MedRec. According to the results after applying Bonferroni test, a better perception level of MedRec was observed in those with more experience years (p < 0.001); higher age groups (p < 0.001); holding master or pharm D (p < 0.001); clinical pharmacists (p < 0.001); pharmacists with previous training or studied a course in the university (p < 0.001); pharmacists working in Dubai Emirate and pharmacists practicing in private teaching hospitals in addition to governmental hospitals (Tables 9, 10).

Post hoc analysis using pairwise comparison shows a statistically significant difference in the perception between the age of [20 to below 30] and [30 to below 40] (p < 0.001), [20 to below 30] and [40 and above] (p = 0.002). The test also shows a statistically significant difference in the perception between the years of experience [< 5 years] and [5–< 10 years] (p = 0.002), [< 5 years] and [≥ 10 years] (p < 0.001). A statistically significant difference is presented between perception and qualification [bachelor] and [master or Pharm D] (p < 0.001). The test shows a statistically significant difference in the perception between the type of hospitals [private non-teaching] and [private teaching] (p = 0.004), [semi-government] and [government] (p = 0.002), [semi-government] and [private teaching] (p < 0.001). Working Emirates is also a factor that affects the perception as the test shows a statistically significant difference in the perception between those working in Abu-Dhabi and Dubai Emirates (p < 0.001).

#### Barriers

Factors that have an impact on the participants’ perceived barriers to implementing MedRec can be seen in Tables 9 and 10. Post hoc analysis using pairwise comparison showed a statistically significant difference in the barrier between the pharmacy site of work [outpatient pharmacy] and [pharmacy on duty] (p < 0.001).

## Discussion

It is indisputable that reducing medication errors is a matter of concern for every healthcare system^34^. Different strategies have been proposed and implemented to overcome the threat of these errors^4^. MedRec is an important process that can detect medication discrepancies, minimize medication errors, and hence enhance safe medication use^5,35,36^. Although it is a shared responsibility of all healthcare providers, given pharmacists’ expertise in medicines, pharmacist-led medicines reconciliation should be recognized for its accuracy in completing patients’ medication histories, determining and reconciling medication discrepancies, and suggesting the necessary interventions^37–39^.

The UAE consists of seven Emirates wherein its healthcare system has witnessed rapid growth in its capacity and quality^40^. The Federal Ministry of Health (MOH) is regulating the health system in the Northern Emirates in addition to regulating the high-level functions in all Emirates. Abu-Dhabi and Dubai Emirates have their own health regulations^41^. It has been reported that the quality and cost of services vary between Emirates as less development is realized in Northern Emirates compared to Dubai and AbuDhabi^42^. There are no studies conducted in the UAE to measure the hospital pharmacist’s knowledge, practice and perceptions towards MedRec and to address the main barriers that may render them to practice it, hence this study was performed.

The majority of respondents are confident that they have enough skills to apply MedRec and take “the best possible medication history” from the patients in spite of denying studying a related course in the university. More than half of the pharmacists received a training or attending a workshop related to MedRec and this could explain the high confident level expressed on their skills. Moreover, this study included clinical pharmacists and pharmacists with high qualifications which could increase the confident level. However, the majority of participants admitted that they need more training programs related to MedRec. Our results are in line with Katoue et al. study in Kuwait which indicates that 90% of their hospital pharmacists attended formal training on MedRec while there is a need for more training to practice this process^43^. The same researchers realized this need and they developed a simulation-based workshop to hospital pharmacists in Kuwait^44^. The necessity to train the pharmacists have been recognized globally and different associations published MedRec toolkits that can be utilized to train pharmacists and other healthcare providers^45–47^.

The majority of participants have to implement MedRec to all admitted patients as it is part of their job descriptions. Surprisingly, although most of respondents are aware about MedRec policy in their hospitals, almost one-third of them implemented MedRec for less than five patients per week. These contrary results could be justified by saying that addressing policies and job descriptions are not enough to obligate pharmacists to practice as there could be some barriers that need to be investigated to resolve this mystery. This study revealed that limited role of pharmacists is witnessed in practicing MedRec. These results are consistent with a Jordanian study which contributed lack of awareness among hospital pharmacists to their modest role in the process^27^.

Results of this study show that participants know basic information related to MedRec like its definition and objectives but faced some difficulties in answering “How” questions. This could be due to the fact that majority of participants didn’t study about MedRec in the university. When compared to other studies conducted, low awareness level of the concepts of MedRec and medication safety were addressed^16,48^. Furthermore, a recent published Nigerian study evaluated hospital pharmacists’ knowledge on MedRec as “poor”^49^ whereas a mixed method designed study conducted in US reported lack of knowledge among healthcare providers about the process of MedRec^50^. Interestingly, Lemay J et al. reported higher knowledge of MedRec among hospital pharmacists compared to physicians^21^. Although all the participants in the current study are pharmacists, yet when asked who is the most suitable healthcare provider to take “the best possible medication history”, the minority answered “the pharmacist” while the majority selected “All of the above choice” which include physicians, pharmacists and nurses. MedRec process requires collaboration of interdisciplinary team and every healthcare provider can indeed take part in it, but when it comes to medication history, pharmacists are best suited due to their unique expertise in medications^30,51,52^. A Belgium study was done to compare medication histories acquired by pharmacists versus physicians in emergency department found that pharmacists took medication histories more accurately than physicians and they supported pharmacist role in this step^53^.

The current study revealed that clinical pharmacists, previously trained pharmacists, experienced pharmacists and those with higher educational levels have significantly better knowledge about MedRec than other groups. In contrary, gender; country of graduation and working Emirates did not influence participants’ knowledge. This conforms to the study done by Aje et al., who reported qualification but not gender influenced pharmacists’ knowledge on MedRec^49^. These results confirm that the current practicing pharmacists had graduated from curricula that were not focused on clinical aspects. They also highlight the privilege of completing the educational level and being specialized in clinical pharmacy field which can provide the essential knowledge to deliver services that can improve patients’ clinical outcomes. Besides, as knowledge is cumulative process, it can be consolidated by having more experience where there are more interactions with patients in the daily practice.

The overall perception level about MedRec implementation among participants is very high. Most of the participants agreed that this service should be provided to all patients. They also perceived that they can be integrated with other healthcare providers, providing support and advice for medical team as well as for patients and their families. These results are compatible with many published studies that showed high perception level among pharmacists towards MedRec process^20,21,27^. Factors that significantly influenced pharmacists’ perception level are years of experience, age, qualifications, clinical pharmacy education, training on MedRec, and working Emirate. Again, gender and country of graduation did not have an effect on pharmacists’ perception level. A study conducted in Jordan revealed that postgraduate and experienced pharmacists possess higher perception level than others^27^.

Many factors can affect the practice of MedRec. In this study, the vast majority of participants agreed on that the 6 listed items are barriers that prevent them from practicing MedRec. The main barrier that received the highest agreement level is lack of training, followed by lack of knowledge. Additional barriers include: lack of corporation and communication among healthcare providers, lack of time and lack of access to patients’ medical records. These results are line with what had been mentioned in the literature, for instance, several studies conducted in GCC countries addressed the obstacles towards implementing this process and they concluded that lack of knowledge, lack of policy, lack of time, difficulties in accessing patients’ files, lack of agreement among healthcare providers, patients’ barriers are the main obstacles towards practicing MedRec^43,54,55^. Other countries including US^50^, Brazil^56^, and Ireland^57^ reported similar barriers. The current study illustrates that the barriers experienced by the pharmacists are influenced by their educational level, pharmacy working sites and if they are previously trained. Identifying the obstacles will aid in improving the practice by suggesting certain recommendations. Many researchers studied the key factors to enhance the practice of MedRec and they concluded that providing ongoing education and training to healthcare professionals, fostering an effective communication among them, their propensity for change and support from management are the key elements to improve the practice^58–60^. Additional factors recorded in the literature include using standardized tools in MedRec process and knowing the exact duties and tasks for each healthcare provider^61–63^. It is important to highlight the importance of incorporating interprofessional education in the universities besides focusing on strategies to improve patient safety in training and theoretical courses.

## Strengths and limitations of the study

This study presents a comprehensive work assessing the role of pharmacists in MedRec in UAE and addressing important aspects of patient safety and healthcare quality. The study addresses an existing gap in the literature by focusing on the involvement of pharmacists in MedRec in UAE. It achieves this by evaluating their knowledge, current practices, and perceptions—a domain that has received limited research attention. It also sheds light on the challenges faced by pharmacists and emphasizes the potential for improving patient safety through structured systems and defined roles. To the best of authors’ knowledge, this research is the first of its nature conducted in UAE to assess hospital pharmacists’ knowledge, practice, perception, and perceived barriers towards implementing MedRec where hospital management and other concerned authorities can extract useful information from this work. Additionally, it is a nationwide study that includes hospital pharmacists for all Emirates and a stratified sampling method was used to assure participants proportional to the overall population.

Besides these strong points, this study has some limitations which could be considered in future research. The study used a self-administered questionnaire that has a close-ended question that limited the responses to select from the available options where some important aspects could be missed. For instance, only six barriers are listed to the participants and there could be some other obstacles that are unaddressed. Moreover, this study included diverse hospital settings within the healthcare system in UAE which could make the inference on the current practice of MedRec quite difficult. Also, the researchers did not succeed to achieve the required sample size as only 88.8% had been reached. Furthermore, the hospital pharmacists were approached through their individual pages in LinkedIn platform while there could be other pharmacists who do not use this platform and they are eligible to be included in the study.

## Recommendations

The study recommends that providing hospital pharmacists with comprehensive training and educational workshops on MedRec is essential for improving patient safety and the overall quality of healthcare. The focus of these training courses should be on the technique and the steps of MedRec to perform it accurately and efficiently. It also emphasizes continuous learning and interdisciplinary collaboration. Furthermore, it suggests incorporating more hands-on training on MedRec in pharmacy curricula. This will help students to develop the necessary skills and confidence to perform MedRec accurately and efficiently when they are practiced pharmacists.

Positively, the perception of pharmacists and their confidence level in leading the process is very high in this study indicating a strong commitment to be actively involved in this process. The more confident pharmacists are more likely to take proactive roles and this suggests having clear and well-defined hospital policies that outline the roles, responsibilities, and expectations for pharmacists in MedRec. Clear policies can also enhance collaboration among healthcare team members, including pharmacists, nurses, and physicians as this collaboration is essential for accurate and comprehensive MedRec.

As the study design is quantitative in nature, using qualitative or mixed-method research designs is recommended in future research. This can provide a deeper understanding and better recommendations for improving MedRec practices among pharmacists.

## Conclusions

As a conclusion, this study revealed that pharmacists expressed different levels of knowledge but showed a positive perception and willingness to practice MedRec. While the role of pharmacists in MedRec process is recognized worldwide, it is important to establish a system that defines their roles in UAE hospitals. The Department of Health Services in UAE has adopted number of plans to enhance medication therapy management services, however, pharmacists’ expanding duties and pharmaceutical care are not really ideal from a practical standpoint. Moreover, hospital pharmacists have highlighted several barriers to practicing MedRec, such as a lack of knowledge and training in this field. It is crucial to offer a continuing education program using various educational approaches to enhance MedRec practice among hospital pharmacists.

## Data Availability

The datasets used and/or analyzed during the current study are available from the corresponding author upon reasonable request.
